# Supplementary material for: The FLUXCOM ensemble of global land-atmosphere energy fluxes
Source: Sci Data. 2019 May 27;6:74. doi: 10.1038/s41597-019-0076-8 (PMC6536554; doi:10.1038/s41597-019-0076-8)
Supplement: Supplementary file 2 — Supplementary Information [file 41597_2019_76_MOESM2_ESM.docx]

**Supplementary Information 1**

**Contributions to ensemble spread**

To assess how the different factors, energy balance closure (EBC) variants, machine learning methods (MLM), and meteorological forcing choices (METEO) contribute to the ensemble spread we measure the ensemble spread by only varying one factor at a time and keeping the other two fixed. This can be taken as a sensible measure of sensitivity of the factor under analysis. Specifically, taking EBC uncertainty as guiding example, we calculate for each combination of METEO and MLM the median absolute deviation (MAD) for the ensemble members where METEO and MLM are kept the same:

$\sigma_{MLMi, METEOj}^{EBC}=mad(E_{MLMi,METEOj}^{EBCall})$ (Eq.1)

Where i and j are indices for MLM and METEO respectively, and E denotes the set of ensemble members. Equation (1) is applied to each combination of MLM and METEO resulting in nMLM × nMETEO values for σ^EBC^ over which we take the mean and standard deviation. A large standard deviation reflects large interaction effects with the other two factors (here MLM and METEO). The mean and the standard deviation of σ^EBC^ are then normalized by the ensemble spread (mad) of the full ensemble to get the relative importance (RI) of EBC with an error bar:

${RI}_{\mu}^{EBC}=\frac{\sigma_{\mu}^{EBC}}{mad(E)} and {RI}_{\sigma}^{EBC}=\frac{\sigma_{\sigma}^{EBC}}{mad(E)}$ (Eq.2)

The advantage of this method over an ANOVA is that we can be consistent with our definition of ensemble spread (mad) and do not require assumptions of normality. A disadvantage of this method is the calculation of mad in Eq.1 when only few realizations for a factor are available (e.g. here three for EBC). Please note that RI_µ_ for the three factors do not add to 1. We applied this way of assessing the contributions of different factors to ensemble spread to the global and continental mean annual energy fluxes (Fig. SI-1.1).


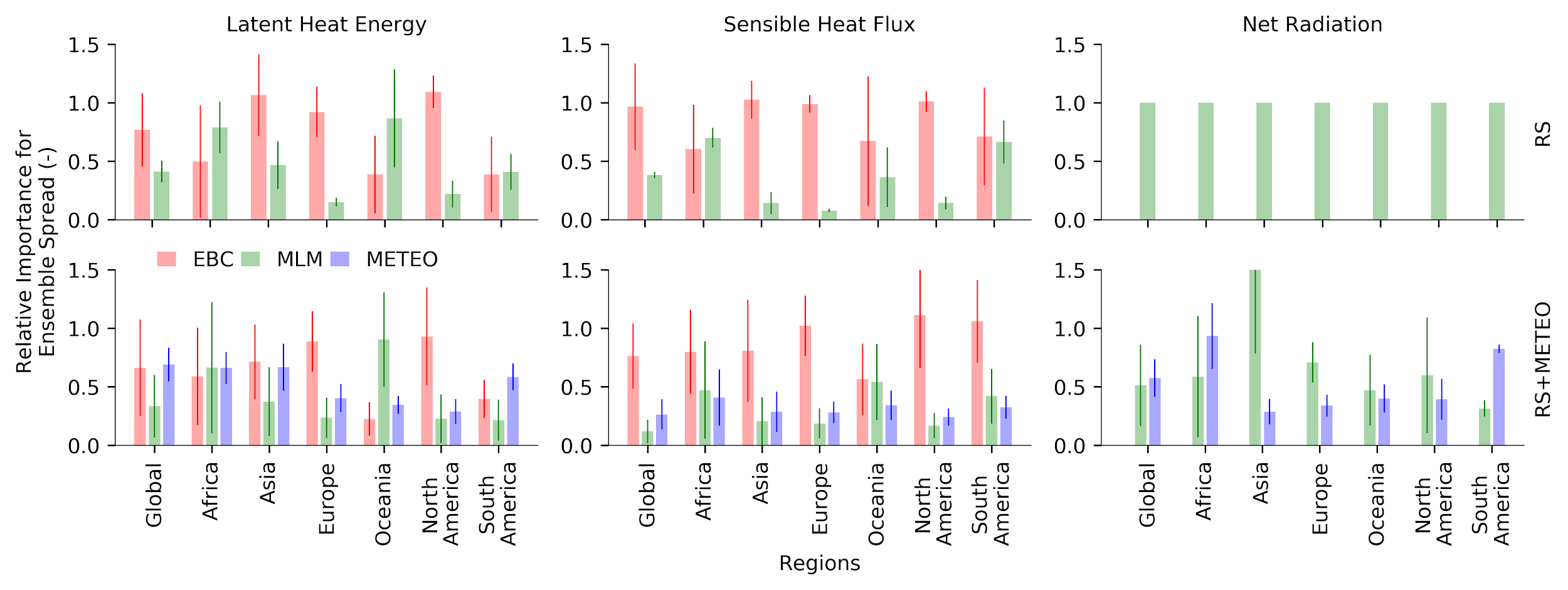


Figure S1: Relative importance of the factors energy balance correction (EBC), machine learning method (MLM), and meteorological forcing (METEO) for the ensemble spread of global and continental energy fluxes. Top row: for FLUXCOM-RS; bottom row: FLUXCOM RS+METEO.

The contributions of energy balance closure (EBC) uncertainty, machine learning method (MLM), and meteorological forcing (METEO) to the ensemble spread of global and continental mean annual energy fluxes are all sizeable and their relative importance varies by continent. Global latent heat flux uncertainty of the FLUXCOM RS ensemble is dominated by EBC while METEO uncertainty is of similar magnitude in RS+METEO. In the FLUXCOM RS ensemble of LE EBC uncertainty is usually larger than MLM uncertainty but there are important exceptions like Africa and Oceania where MLM uncertainty dominates. This is likely due to comparatively poor constraints by FLUXNET stations in similar water limited systems. Contributions of METEO uncertainty to the FLUXCOM RS+METEO ensemble spread for LE are particularly large for Global, Africa, Asia, and South America. Global and continental sensible heat flux uncertainty are dominated by EBC in both RS and RS+METEO setups. The larger importance of EBC for H uncertainty compared to LE uncertainty is because mean H is typically smaller than mean LE. Thus the “residual approach” of energy balance closure correction (H_RES_ & LE_NONE_) adds a relatively larger value to the measured H (compared to LE_RES_ & H_NONE_ for LE). MLM and METEO contribute roughly similarly to FLUXCOM RS+METEO net radiation uncertainty but their relative importance varies by continent.
